# KMT5A-mediated methylation of IRF3 promotes tumor progression through immune suppression

**DOI:** 10.1016/j.isci.2026.116902

**Published:** 2026-07-21

**Authors:** Pengcheng Li, Chengxin Yu, Runshi Xie, Changsheng Huang, Qi Wu, Anyi Liu, Xiaowei She, Mao Li, Zejun Rao, Lang Liu, Guihua Wang, Junbo Hu, Li Sun

**Affiliations:** 1 GI Cancer Research Institute, Tongji Hospital, Huazhong University of Science and Technology, Wuhan 430030, China; 2 Department of Oncology, Tongji Hospital, Huazhong University of Science and Technology, Wuhan 430030, China; 3 State Key Laboratory for Diagnosis and Treatment of Severe Zoonotic Infectious Diseases, Huazhong University of Science and Technology, Wuhan, Hubei Province 430030, China

**Keywords:** Biological sciences, Epigenetics, cancer

## Abstract

Lysine methylation is a critical post-translational modification (PTM) involved in diverse physiological and pathological processes. Interferon regulatory factor 3 (IRF3) plays a pivotal role in antitumor immunity; however, the regulatory mechanisms and functional impact of IRF3 methylation within the tumor microenvironment remain incompletely understood. This study demonstrates that monomethylation of IRF3 at lysine 193(K193) suppresses its phosphorylation-dependent activation. Mass spectrometry-based protein interactome analysis identified lysine methyltransferase 5A (KMT5A) as the key enzyme responsible for IRF3 K193 monomethylation. In colorectal cancer (CRC), aberrantly high expression of KMT5A impaired *in vivo* antitumor immune responses. Mechanistically, KMT5A catalyzes IRF3 monomethylation at K193, which impedes IRF3 phosphorylation and subsequent activation, thereby suppressing the production of type I interferons (IFN-I). Collectively, these findings elucidate KMT5A-mediated IRF3 K193 methylation as a critical regulatory axis promoting tumor immune evasion and progression. Furthermore, IRF3 K193 methylation represents a promising therapeutic target for CRC intervention.

## Highlights

• High KMT5A levels correlate with lower intratumoral CD8^+^ T cell infiltration
• KMT5A mediates monomethylation of IRF3 at K193
• KMT5A-mediated IRF3 methylation suppresses its phosphorylation and activation
• KMT5A inhibitor UNC0379 improves colorectal cancer immunotherapy response

## Graphical abstract

**Figure:**
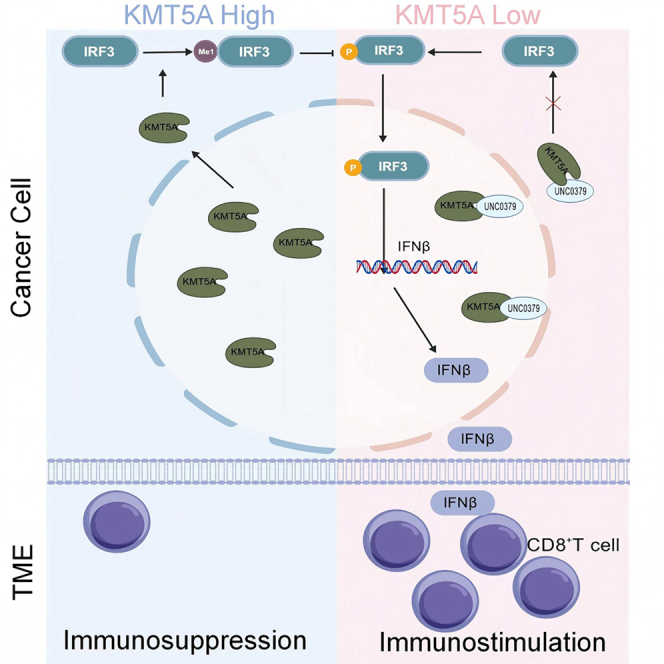

## Introduction

Interferon regulatory factor 3 (IRF3) is a central transcription factor (TF) widely expressed in mammalian cells, playing dual roles in antiviral innate immunity and tumor suppression.^1^ In unstimulated cells, IRF3 resides predominantly in the cytoplasm. Upon activation of pattern recognition receptors (PRRs), it undergoes phosphorylation at its C-terminal domain by TBK1/IKKε, leading to nuclear translocation and initiation of type I interferon (IFN-I) production.^2^ Beyond its canonical antiviral function, IRF3 acts as a tumor suppressor in colorectal cancer (CRC) through multiple mechanisms, including inhibition of β-catenin nuclear accumulation,^3^ and suppression of TGF-β/Smad-mediated regulatory T cell differentiation and epithelial-mesenchymal transition (EMT).^4^ The functional regulation of IRF3 involves multiple post-translational modifications (PTMs), with phosphorylation-dephosphorylation cycles constituting the primary activation-inactivation switch,^5^^,^^6^ while acetylation and SUMOylation further fine-tune its transcriptional activity.^7^^,^^8^

Emerging evidence highlights protein methylation—particularly lysine methylation—as a critical regulatory mechanism influencing oncogenic signaling pathways.^9^^,^^10^ This evolutionarily conserved modification, catalyzed by lysine methyltransferases (KMTs), impacts diverse cellular processes through both histone-dependent chromatin remodeling and direct modulation of non-histone proteins.^11^^,^^12^ Notable examples include SET9-mediated methylation of p53 that enhances tumor suppressor activity,^13^and EZH2-dependent methylation of MAD3 that promotes metastatic progression.^14^ Recent discoveries extend this paradigm to metabolic regulation, with SETDB1-mediated methylation of AKT and MCT1 driving tumor initiation and progression.^15^^,^^16^ These findings collectively position lysine methylation as a crucial oncogenic modification and potential therapeutic target.

Our study uncovers a novel methylation-dependent regulatory mechanism controlling IRF3 activity in colorectal carcinogenesis. We demonstrate that the lysine methyltransferase KMT5A directly interacts with IRF3, catalyzing mono-methylation at lysine 193 (K193me1). This modification impedes TBK1/IKKε-mediated phosphorylation, thereby suppressing IRF3 nuclear translocation and transcriptional activation. The KMT5A-IRF3 axis significantly reduces IFN-I production and attenuates antitumor immunity, establishing a mechanistic link between IRF3 methylation status and CRC progression. Our findings identify K193-methylated IRF3 as both a prognostic biomarker and a promising therapeutic target for CRC intervention.

## Results

### KMT5A interacts with IRF3 and is associated with CRC

The comprehensive understanding of the enzymatic system regulating IRF3 PTMs remains incomplete. To systematically identify IRF3-interacting regulatory factors, we performed co-immunoprecipitation (coIP) assays in HEK293T cells overexpressing FLAG-tagged IRF3, followed by mass spectrometry analysis of purified protein complexes (Table S1). This approach revealed lysine methyltransferase 5A (KMT5A/SETD8), a histone H4K20-specific monomethyltransferase,^17^ as a novel binding partner of IRF3, suggesting a potential role for KMT5A in the functional regulation of IRF3.

**Figure 1:**
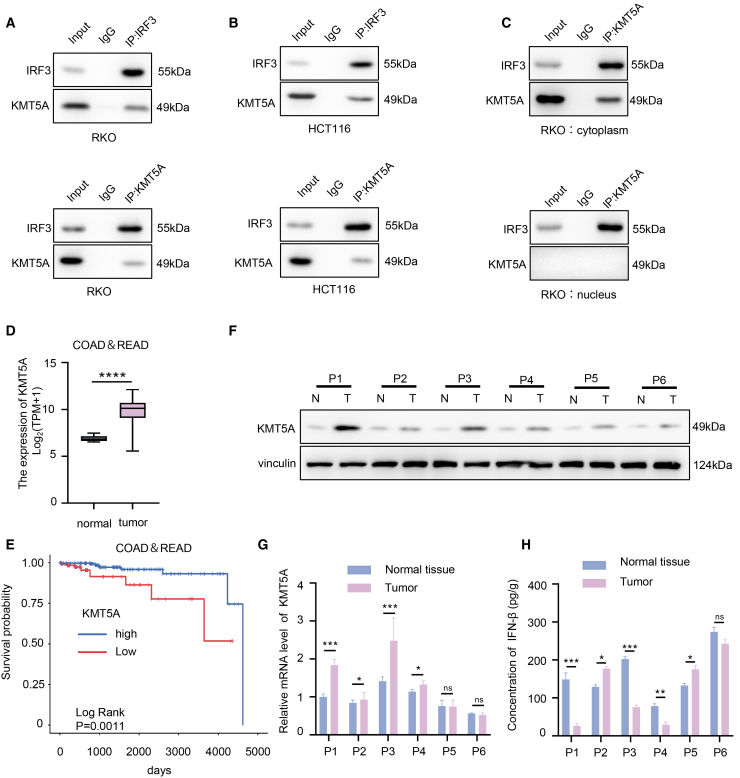
KMT5A interacts with IRF3 and is associated with colorectal cancer (A and B) Whole-cell lysates (WCL) from RKO and HCT116 cells were collected for IP using anti-IRF3 or anti-KMT5A antibodies, followed by IB analysis. (C) Cytoplasmic and nuclear proteins from RKO were collected for IP using anti-KMT5A antibodies, followed by IB analysis. (D) Boxplots were used to compare KMT5A mRNA expression in COAD and READ, with Student’s two-tailed *t* test, *p* < 0.0001. (E) Disease-specific survival analysis of TCGA-COAD and READ based on KMT5A expression levels. (F) Protein expression of KMT5A was analyzed by IB in colorectal tumor and paired adjacent normal tissues. (G) mRNA was extracted from colorectal tumors and paired adjacent normal tissues, and KMT5A mRNA levels were analyzed by qPCR. (H) The levels of IFN-β colorectal tumor and paired adjacent normal tissues were measured using ELISA. For (D)–(H) statistical analysis was performed using one-way ANOVA followed by Tukey’s post-hoc test. Data are presented as mean ± SD. ns *p* > 0.05, ∗*p* < 0.05, ∗∗*p* < 0.01, ∗∗∗*p* < 0.001. All immunoblotting experiments were performed independently three times with similar results.

Reciprocal coIP experiments using IRF3- and KMT5A-specific antibodies confirmed this interaction (Figures 1A and 1B). To determine the subcellular localization of the interaction between KMT5A and IRF3, we conducted cytoplasmic and nuclear protein separation experiments, followed by coIP using a KMT5A-specific antibody. Our findings indicate that KMT5A is not solely restricted to the nucleus but is also detectable in the cytoplasm. Moreover, the coIP results demonstrated that the interaction between KMT5A and IRF3 occurs predominantly in the cytoplasmic compartment, with minimal association observed in the nucleus (Figure 1C). This pattern aligns with the established cytoplasmic localization of IRF3 under unstimulated cellular conditions.

Given emerging evidence of KMT5A’s oncogenic potential, we investigated its clinical relevance in CRC. Mining of data from The Cancer Genome Atlas (TCGA) revealed a significant upregulation of KMT5A in CRC cohorts, and its elevated expression levels correlated with reduced overall survival (Figures 1D and 1E). To further assess the role of KMT5A in CRC, clinical validation using paired tumor/adjacent normal tissues from CRC patients demonstrated significantly higher KMT5A expression at both mRNA and protein levels in tumor tissues compared to normal counterparts (Figures 1F and 1G). Notably, tumors exhibiting high KMT5A expression showed a trend toward downregulation of IFN-β, a critical antitumor cytokine (Figure 1H). These results indicate that KMT5A interacts with IRF3 and may promote tumor progression by suppressing the production of IFN-I.

### KMT5A affects CD8^+^ T cell infiltration in colorectal tumors

**Figure 2:**
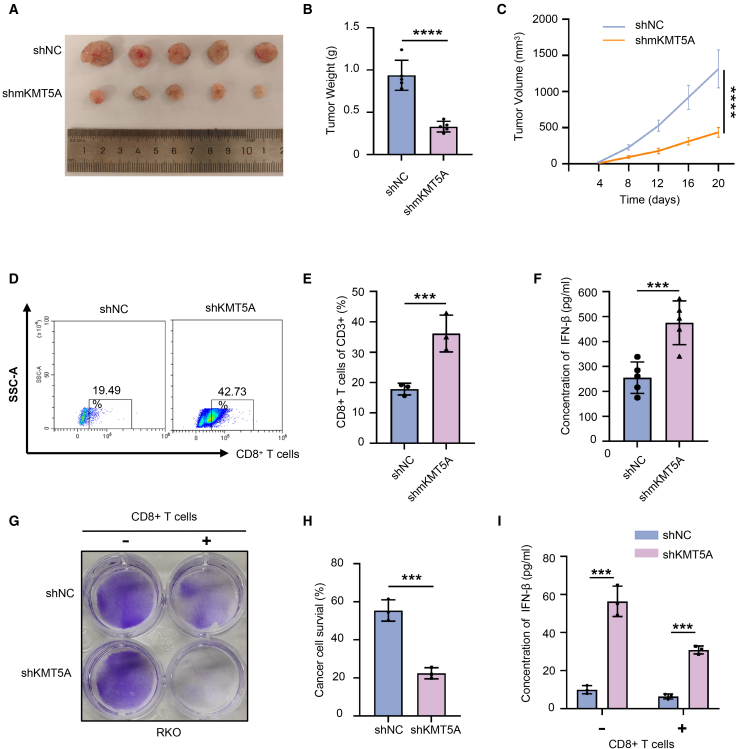
KMT5A affects immune infiltration in colorectal tumors (A) To establish tumor models, MC38 cells with either control or mKMT5A knockdown via shRNA were injected subcutaneously into C57BL/6 mice. (B and C) Tumor mass and volume derived from the experiments in (A) were quantified. (D and E) Flow cytometry was conducted to analyze CD8^+^ T cell infiltration in the tumors from (A). (F) The levels of IFN-β in the tumors from (A) were measured using ELISA. (G and H) Activated T cells co-cultured with pre-treated RKO cells. Representative images (G) and statistical analysis (H) are shown. (I) The levels of IFN-β in the cell culture supernatant from (G) were measured using ELISA. For (B), (E), (F), (H), and (I) statistical analysis was performed using one-way ANOVA followed by Tukey’s post-hoc test. For (C), two-way ANOVA with Tukey’s post-hoc test was used. Data are presented as mean ± SD. ∗∗∗*p* < 0.001, ∗∗∗∗*p* < 0.0001.

We developed an *in vivo* tumor model using C57BL/6 mice. MC38 CRC cells were either transduced with control short hairpin RNA (shNC) or subjected to mKMT5A knockdown via shRNA. These cells were then injected subcutaneously into the mice to observe the effects on tumor growth. Strikingly, 20 days after injection, we observed a significant inhibition of tumor growth in the mKMT5A-knockdown group compared to the control group (Figures 2A–2C). This suggests that reducing KMT5A levels hampers the tumor’s ability to proliferate and grow. Histopathological evaluation of resected tumors revealed dual microenvironmental modifications: increased CD8^+^ T cell infiltration (Figures 2D and 2E), and elevated IFN-β levels in KMT5A-deficient tumors (Figure 2F).

We conducted T cell-mediated tumor cell killing assays by co-culturing activated human primary T cells with either RKO wild-type (RKO WT) cells or RKO KMT5A-knockdown (RKO shKMT5A) cells. The results demonstrated that KMT5A significantly inhibited the killing activity of T cells against tumor cells, whereas knockdown of KMT5A enhanced the cytotoxic ability of T cells (Figures 2G and 2H). Additionally, we measured the concentration of interferon-beta (IFN-β) in the cell supernatant (Figure 2I). These findings strongly suggest that KMT5A inhibits the secretion of IFN-β and ultimately promotes cancer cell growth by suppressing T cell function.

### KMT5A is associated with the IFN-I pathway

**Figure 3:**
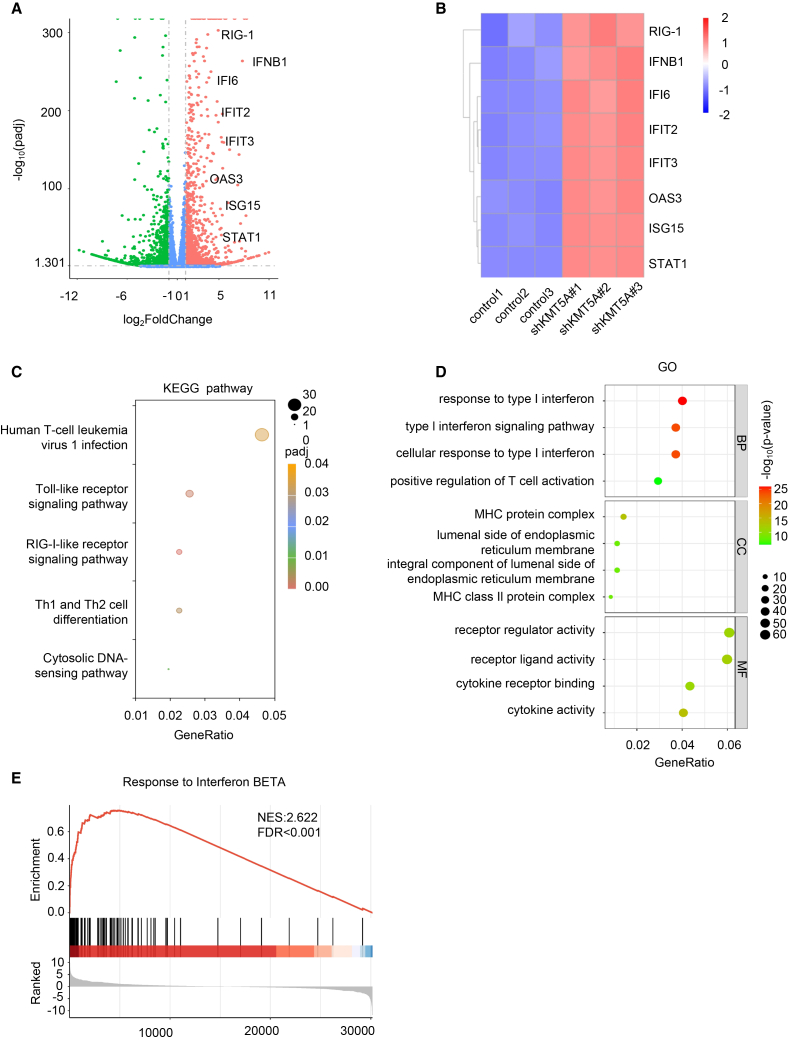
KMT5A is associated with the type I interferon pathway Control and KMT5A knockdown RKO cells were subjected to RNA sequencing (RNA-seq) analysis. (A) Volcano plot depicting differentially expressed genes, with upregulated genes in the knockdown group compared to the control highlighted in red. (B) Heatmap illustrating the expression patterns of the differentially expressed genes. (C) KEGG pathway enrichment analysis of significantly altered pathways in KMT5A-knockdown cells based on transcriptomic data. (D) Gene Ontology (GO) analysis of significantly differentially expressed genes in KMT5A-knockdown cells, categorizing them into biological processes (BP), cellular components (CC), and molecular functions (MF). (E) Gene set enrichment analysis (GSEA) of gene sets related to the upregulation of interferon BETA-associated pathways.

To elucidate the potential oncogenic mechanisms underlying KMT5A activity, we performed comprehensive RNA-sequencing analysis of RKO cells with targeted KMT5A knockdown (shKMT5A#1, #2, #3) compared to non-targeted control cells (shNC). The transcriptomic profiling revealed that KMT5A depletion significantly upregulated a range of immune-related genes, including IFNB, IRF3, IFI6, OAS3, and STAT1. These genes are predominantly implicated in antiviral immunity, anti-tumor responses, and the regulation of IFN-I secretion (Figures 3A and 3B; Table S2). Further analysis using the Kyoto Encyclopedia of Genes and Genomes (KEGG) pathways showed that the knockdown of KMT5A enhanced the expression of key components in the Toll-like receptor signaling pathway, RIG-I-like receptor signaling pathway, helper T cell differentiation, and the cytosolic DNA-sensing pathway, highlighting a broad activation of innate immune signaling cascades.

Moreover, Gene Ontology (GO) enrichment analysis pointed to the upregulation of multiple genes involved in IFN-I response and signaling pathways, as well as genes associated with the positive regulation of T cell activation. These changes were accompanied by an increased expression of gene products localized to the endoplasmic reticulum, Golgi apparatus, and major histocompatibility complex (MHC), which are crucial for the modulation of cytokine production and receptor-ligand interactions, further emphasizing a systemic enhancement of immune activity (Figure 3D).

Gene set enrichment analysis (GSEA) further corroborated these findings, revealing a marked enrichment of the IFN-β signaling pathway among the upregulated gene sets in KMT5A-deficient cells (Figure 3E). Collectively, these data suggest that KMT5A acts as a negative regulator of IFN-I production and release.

### KMT5A inhibits phosphorylation activation of IRF3

**Figure 4:**
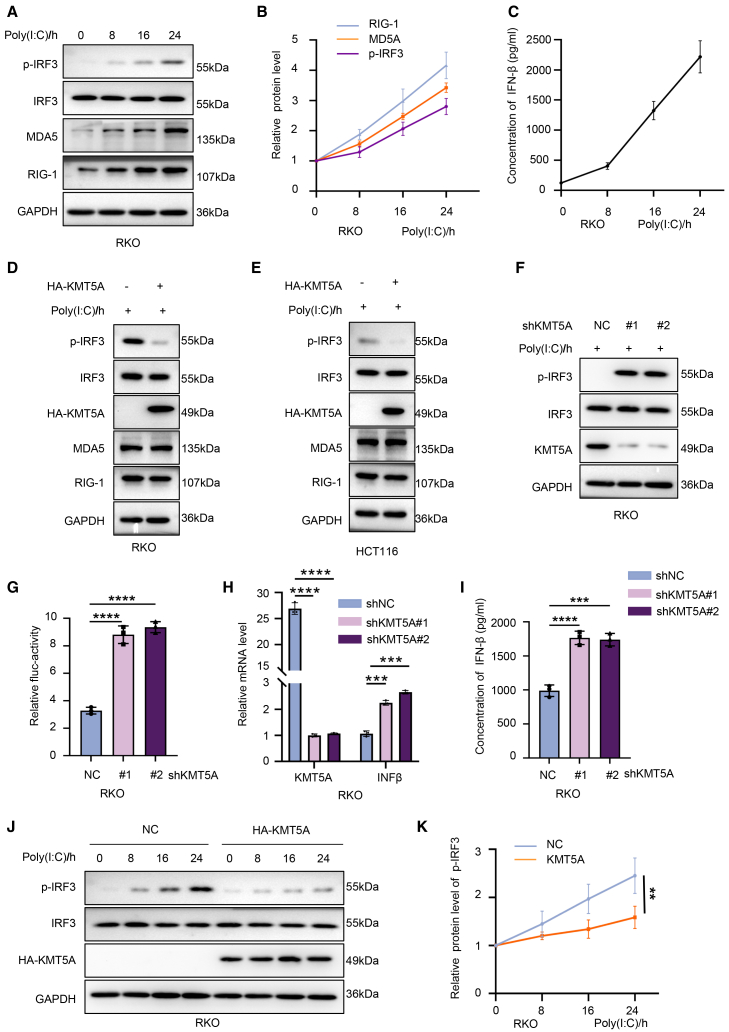
KMT5A inhibits phosphorylation activation of IRF3 (A) RKO cells were treated with poly(I:C) and protein samples were collected at 0, 8, 16, and 24 h for Western blot (WB) analysis. (B) Quantification of p-IRF3, RIG-1, and MDA5 protein levels relative to control in the samples collected in (A). (C) IFN-β secretion levels at different time points from (A) were measured using ELISA. (D, E) RKO and HCT116 cells transfected with either an empty vector or a KMT5A overexpression plasmid were treated with poly(I:C), followed by WB analysis of whole-cell lysates. (F) RKO cells transduced with control shRNA (shNC) or KMT5A-specific shRNAs (#1 and #2) were treated with poly(I:C) and analyzed by WB using whole-cell lysates. (G) RKO cells from (F) were co-transfected with the IFN-β-Luc reporter plasmid and pRL-TK plasmid, and after 24 h, luciferase activity was measured using a dual-luciferase assay kit. (H) The relative mRNA levels of KMT5A and INF-β in RKO cells from F were quantified using qPCR. (I) IFN-β levels in the RKO cells from (F) were measured using ELISA. (J) RKO cells, either wild-type or stably expressing HA-KMT5A, were treated with poly(I:C) and whole-cell lysates were collected at 0, 8, 16, and 24 h for WB analysis. (K) Quantification of p-IRF3 protein levels relative to control in samples collected in (J). For (G)–(I), statistical significance was determined using one-way ANOVA followed by Tukey’s post-hoc test. For (K), two-way ANOVA with Tukey’s post-hoc test was used. Data are presented as mean ± SD. Statistical significance is indicated as ∗∗∗*p* < 0.001, ∗∗∗∗*p* < 0.0001. All western blot analyses were performed independently three times, yielding consistent results.

To dissect KMT5A’s regulatory role in IRF3-mediated IFN-β production, we employed polyinosinic:polycytidylic acid (poly(I:C), 5 μg/mL)—a dsRNA mimic activating RIG-I-like receptors (RLRs)—in colorectal carcinoma models (RKO and HCT116 cells). Time-course analysis (0–24 h post-stimulation) revealed progressive IRF3 phosphorylation increasing within 24 h (Figures 4A–4C; S1A–S1C). As the phosphorylation of IRF3 is indicative of its activation, these results suggest that IRF3 is progressively activated in response to poly(I:C) stimulation. Notably, the upregulation of RIG-1 and MDA5 preceded the activation of IRF3, indicating that poly(I:C) triggers a signaling cascade that initiates with RIG-1 and MDA5, leading to subsequent IRF3 phosphorylation and IFN-β production (Figures 4B; S1B).

To further clarify the influence of KMT5A on this signaling pathway, we overexpressed HA-tagged KMT5A in RKO and HCT116 cells and then treated them with poly(I:C) for 24 h. We observed that overexpression of KMT5A significantly suppressed IRF3 phosphorylation without affecting the expression levels of MDA5 and RIG-1, nor the total protein levels of IRF3 (Figures 4D and 4E). This suggests that KMT5A may regulate IRF3 activation through mechanisms independent of MDA5 and RIG-1 expression or by directly interfering with IRF3 phosphorylation itself.

Conversely, knockdown of KMT5A in both RKO and HCT116 cells using short hairpin RNA resulted in elevated *p*-IRF3 levels, corroborating the inhibitory role of KMT5A on IRF3 phosphorylation (Figures 4F; S1D). To further validate these observations, we co-transfected control and KMT5A-knockdown cells with a luciferase reporter plasmid driven by the IFN-β promoter, along with a Renilla luciferase plasmid (pRL-TK), 12 h before poly(I:C) treatment. Following poly(I:C) stimulation, we assessed luciferase activity as a measure of IFN-β promoter activation. Consistent with the increased p-IRF3 levels, KMT5A-knockdown cells exhibited significantly higher IFN-β promoter-driven luciferase activity compared to control cells, reflecting an enhanced activation of the IFN-β promoter (Figures 4G; S1E). Endogenous IFN-β levels, as determined by ELISA, further confirmed this trend, demonstrating higher IFN-β production in KMT5A-deficient cells (Figures 4I; S1G).

Finally, we evaluated the kinetics of IRF3 activation in response to poly(I:C) in cells overexpressing HA-KMT5A. We found that IRF3 activation was notably delayed in these cells, suggesting that KMT5A overexpression imposes a temporal restraint on the activation of IRF3 (Figures 4J and 4K; S1H, S1I). While KMT5A did not completely block IRF3 activation, its expression clearly slowed down the process, thereby modulating the amplitude and timing of the IFN-β response. These findings highlight a nuanced role for KMT5A in the regulation of IRF3-mediated antiviral signaling, potentially contributing to its broader impact on immune modulation and tumorigenesis.

### KMT5A induces mono-methylation of IRF3 at K193

To further elucidate the molecular mechanisms by which KMT5A inhibits IRF3 phosphorylation and activation, we focused on the critical regulatory role of PTMs, which are known to be pivotal in modulating protein function.^9^ As a histone H4K20 monomethyltransferase, KMT5A extends its methylation activity to non-histone substrates. For instance, KMT5A promotes triple-negative breast cancer metastasis by monomethylating SNIP1 at lysine 301 (K301) to activate the YAP signaling pathway^18^ and drives non-small cell lung cancer progression by methylating CD147 at lysine 234 (K234) to facilitate lactate export.^19^ Given these findings, we hypothesized that KMT5A might exert its regulatory effects on IRF3 phosphorylation and activation through a similar methylation mechanism.

**Figure 5:**
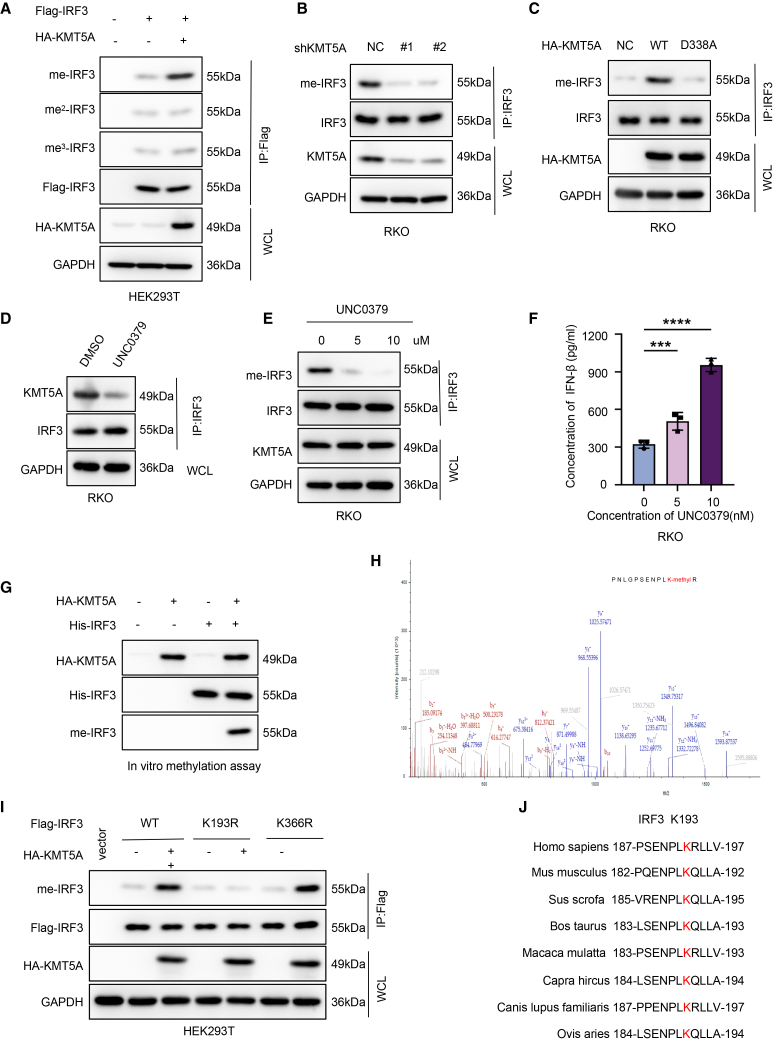
KMT5A induces mono-methylation of lysine 193 on IRF3 (A) HEK293T cells were transfected with FLAG-IRF3 and/or HA-KMT5A plasmids. Whole-cell lysates were collected, followed by IP using anti-FLAG magnetic beads. Subsequent analysis was conducted via IB. (B) Whole-cell lysates were extracted from RKO cells with either control (shNC) or KMT5A shRNA (#1 and #2) silencing. IP was performed using anti-IRF3 antibodies, followed by WB analysis. (C) RKO cells were transfected with HA-KMT5A WT or HA-KMT5A D338A, IP was performed using anti-IRF3 antibodies, followed by WB analysis. (D) RKO cells were treated with either DMSO or UNC0379. IP was performed using anti-IRF3 antibodies, followed by IB analysis. (E) RKO cells were treated with either DMSO or varying concentrations of UNC0379. IP was conducted using anti-IRF3 antibodies, followed by IB analysis. (F) The levels of IFN-β in RKO cells from experiment (E) were quantified using ELISA. Data were analyzed using one-way ANOVA with Tukey’s post-hoc test, presented as mean ± SD. Statistical significance was defined as ∗∗∗*p* < 0.001 and ∗∗∗∗*p* < 0.0001. (G) *In vitro* methylation assays were conducted by incubating purified His-IRF3 with KMT5A in the presence of S-adenosyl-L-methionine, followed by IB analysis. (H) Secondary mass spectrometry results of IRF3 K193 methylation were obtained. (I) HEK293T cells were transfected with FLAG-IRF3 wild-type or mutant plasmids, followed by transfection with either a vector or HA-KMT5A. Whole-cell lysates were collected, and IP was performed using anti-FLAG magnetic beads, followed by IB analysis. (J) Amino acid sequences at the K193 site of IRF3 were compared across different species. All immunoblotting experiments were conducted independently in triplicate, yielding consistent results.

Experimental validation demonstrated that in HEK-293T cells co-transfected with FLAG-IRF3 and HA-KMT5A, immunoprecipitation (IP) assays revealed that KMT5A overexpression significantly enhanced IRF3 monomethylation levels without affecting di- or trimethylation (Figure 5A). Conversely, knockdown of KMT5A in RKO and HCT116 cells markedly reduced IRF3 monomethylation (Figures 5B; S2A). Furthermore, we generated a catalytically inactive KMT5A mutant (D338A),^20^ which abrogated the ability to methylate IRF3 compared to WT KMT5A (Figures 5C; S2B).

To further dissect this interaction, we employed an inhibitor of KMT5A. Currently, the available KMT5A inhibitors remain limited in variety. Research has identified UNC0379 as a highly selective competitive inhibitor of KMT5A^21^(Figure S2D), yet it has not been developed into a clinically approved therapeutic agent. We found that it effectively blocked the binding of KMT5A to IRF3 (Figure 5D). Treatment of RKO and HCT116 cells with increasing concentrations of UNC0379 led to a dose-dependent decrease in IRF3 methylation levels (Figures 5E; S2E). Conversely, IFN-β levels increased in response to UNC0379 treatment (Figures 5F; S2F). An *in vitro* methylation assay confirmed that KMT5A directly catalyzed the methylation of IRF3 (Figure 5G).

Subsequently, we aimed to pinpoint the specific lysine residue on IRF3 that is methylated by KMT5A. We conducted a coIP in HEK293 cells overexpressing FLAG-IRF3, followed by mass spectrometry analysis to identify potential lysine methylation sites. This analysis revealed a single candidate, K193, as a likely methylation target (Figure 5H). Previous studies have shown that NSD3 methylates IRF3 at K366 to enhance antiviral innate immune responses.^12^ Inspired by this, we generated IRF3 plasmids with point mutations at two lysine residues to disrupt their methylation potential and transfected them into HEK293T cells, with or without KMT5A overexpression. Our data demonstrated that only the monomethylation of IRF3 at K193 was specifically dependent on KMT5A (Figure 5I). Importantly, K193 was found to be a conserved methylation site in mammals (Figure 5J).

Collectively, these findings provide compelling evidence supporting our hypothesis that KMT5A specifically induces mono-methylation of IRF3 at K193.

### KMT5A hindering production of IFN-β depends on IRF3 K193 methylation

**Figure 6:**
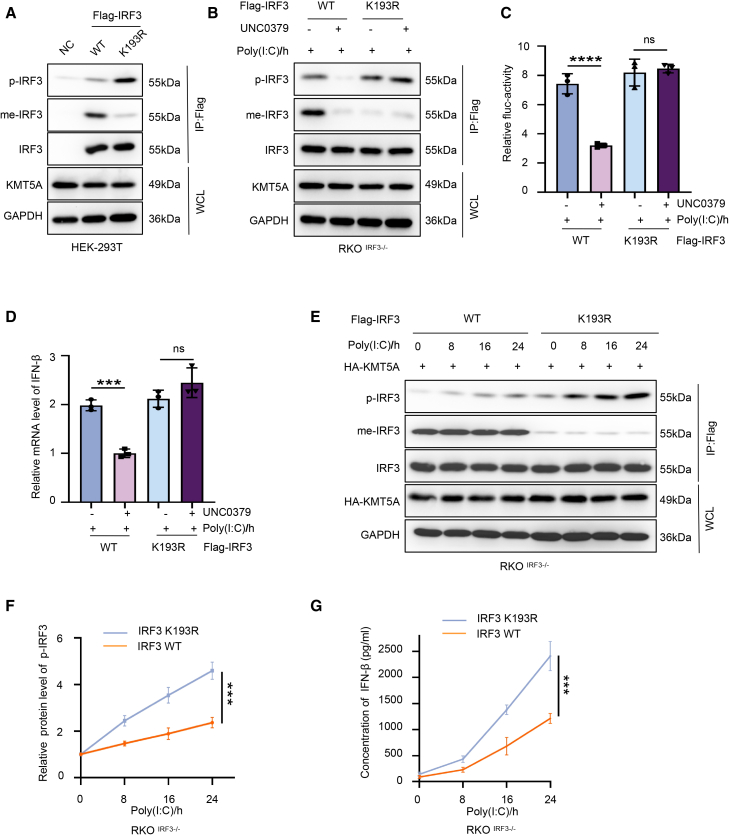
KMT5A hindering production of IFN-β depends on IRF3 K193 methylation (A) HEK293T cells were transfected with vector, FLAG-IRF3 wild-type, or K193R mutant plasmids. Whole-cell lysates were collected, followed by IP using anti-FLAG magnetic beads, and subsequent analysis was performed via IB. (B) KMT5A-knockout RKO cells were transfected with FLAG-IRF3 wild-type or K193R mutant plasmids. The cells were treated with poly(I) or UNC0379, then whole-cell lysates were collected, and IP was conducted using anti-FLAG magnetic beads, followed by IB analysis. (C) RKO cells from experiment (B) were co-transfected with reporter plasmids IFN-β-Luc and pRL-TK. After 24 h, the cells were harvested, and luciferase activity was measured using a dual-luciferase reporter assay kit. (D) The relative mRNA levels of INF-β in RKO cells from experiment (B) were quantified using qPCR. (E) KMT5A knockout RKO cells were transfected with HA-KMT5A and FLAG-IRF3 wild-type or K193R mutant plasmids. Whole-cell lysates were collected, followed by IP using anti-FLAG magnetic beads, and subsequent IB analysis was performed. (F) Relative quantification of phosphorylated IRF3 (p-IRF3) protein was conducted in experiment (E). (G) The relative mRNA levels of INF-β in RKO cells from experiment F were quantified using qPCR. (C) and (D) were analyzed using one-way ANOVA with Tukey’s post-hoc test, with data presented as mean ± SD. (F) and (G) were analyzed using two-way ANOVA with Tukey’s post-hoc test. Statistical significance was defined as ∗∗∗*p* < 0.001, ∗∗∗∗*p* < 0.0001, while ns indicates no statistical difference. All immunoblotting experiments were performed independently in triplicate, yielding consistent results.

Our earlier studies revealed that KMT5A catalyzes monomethylation of IRF3 at the K193 residue, thereby inhibiting its phosphorylation and subsequent activation. However, the precise relationship between these two processes remained unclear. To elucidate this mechanistic link, we transfected HEK293T cells with either WT or mutant IRF3 constructs and stimulated them with poly(I:C)for 24 h, followed by IP analysis using anti-FLAG antibodies. We observed that the K193R mutant, which is deficient in monomethylation at the K193 site, exhibited a significant decrease in monomethylation levels alongside a concomitant increase in phosphorylation levels (Figure 6A), suggesting that monomethylation of IRF3 at K193 restrains its phosphorylation and activation.

To further investigate the functional significance of this modification, we employed the CRISPR-Cas9 system to generate IRF3 knockout (KO) RKO cells (Figure S2C) and subsequently stably expressed the methylation-deficient IRF3 K193R mutant in these IRF3-knockout RKO cells. Consistent with our hypothesis, treatment with the KMT5A inhibitor UNC0379 failed to suppress the phosphorylation and activation of the IRF3 K193R mutant (Figure 6B). We then co-transfected these RKO cells with IFN-β-Luc and pRL-TK reporter plasmids to evaluate IFN-β promoter activity and endogenous IFN-β production. The results demonstrated that neither the activation of the IFN-β promoter nor the production of endogenous IFN-β in IRF3 K193R mutant-expressing cells was affected by UNC0379 treatment (Figures 6C and 6D).

Subsequently, we overexpressed KMT5A in the aforementioned cell lines and stimulated them with poly(I:C)for various time intervals to monitor IRF3 phosphorylation and IFN-β production (Figure 6E). In cells lacking IRF3 K193 monomethylation, the inhibitory effect of KMT5A on IRF3 phosphorylation and IFN-β production was significantly attenuated (Figures 6F and 6G). These findings highlight that the ability of KMT5A to suppress IRF3 activation and the subsequent downregulation of IFN-β production is critically dependent on IRF3 monomethylation at K193.

### UNC0379 could be used as an adjuvant therapy for colorectal tumors

Finally, we sought to investigate the potential of KMT5A as a therapeutic target for CRC. KMT5A is a histone methyltransferase implicated in regulating chromatin structure and gene expression—processes frequently dysregulated in cancer. The synthetically developed small-molecule compound UNC0379 is a selective, competitive inhibitor of KMT5A and exhibits high affinity for this enzyme, making it a promising candidate for targeted therapy. Given these properties, we hypothesized that UNC0379 might be effective in treating CRC, either as a monotherapy or as an adjuvant in combination with immune checkpoint inhibitors.

**Figure 7:**
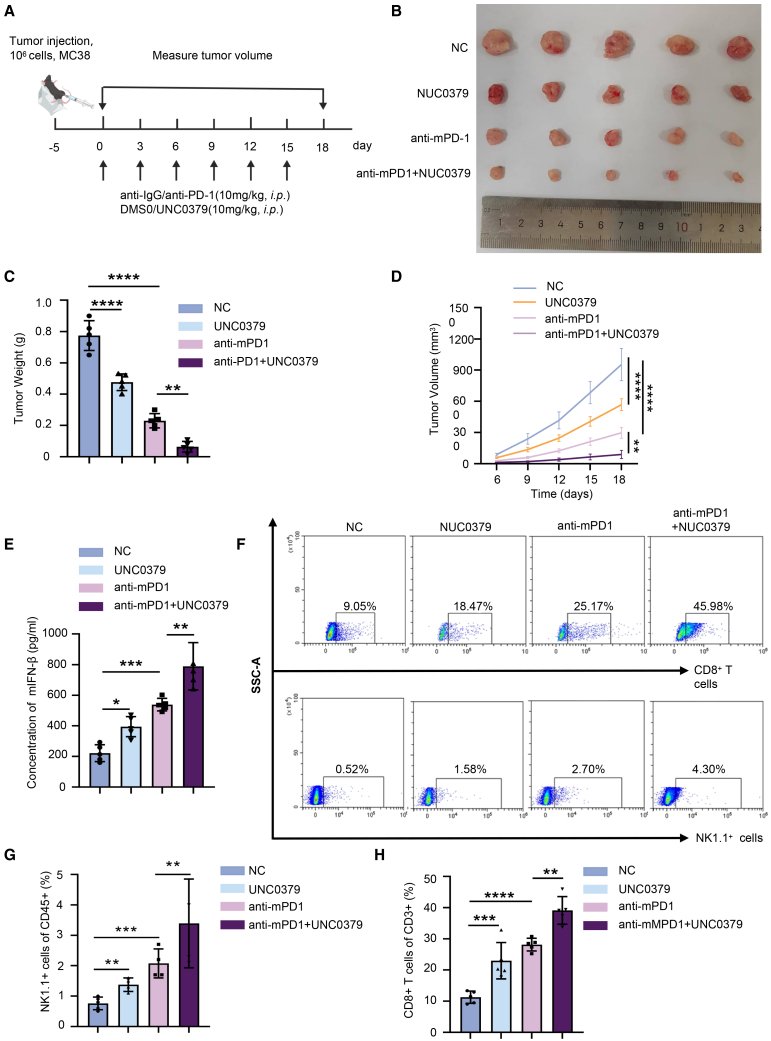
UNC0379 could be used as an adjuvant therapy for colorectal tumors (A) The treatment protocol of anti-PD-1 treatment combined UNC0379 in CRC. (B) C57BL/6 mice were subcutaneously injected with MC38 cells and subsequently treated with PBS, anti-mPD1, UNC0379, or a combination of anti-mPD1 and UNC0379 to establish a tumor model. (C and D) Tumor weight and volume were quantified from the samples obtained in experiment (A). (E) The concentration of IFN-β in the tumors from experiment (A) was assessed using an ELISA assay. (F–H) Flow cytometric analysis was conducted to evaluate the populations of CD8^+^ T cells and NK1.1+ cells in the tumors from experiment (A). For (C), (E), (G), and (H), data were analyzed using one-way ANOVA with Tukey’s post-hoc test. (D) was analyzed using two-way ANOVA with Tukey’s post-hoc test. Data are presented as mean ± SD, with statistical significance indicated as ∗∗*p* < 0.01, ∗∗∗*p* < 0.001, and ∗∗∗∗*p* < 0.0001.

To test this hypothesis, we conducted in vivoexperiments using C57BL/6 mice subcutaneously injected with MC38 CRC cells. The mice were treated with UNC0379(10 mg/kg),^22^ anti–PD-1(10 mg/kg),^23^ or a combination of both agents (Figure 7A). Our results revealed that although UNC0379 alone exerted only a moderate inhibitory effect on tumor growth, its combination with anti-PD-1 significantly enhanced the antitumor response. Notably, the combination therapy substantially inhibited tumor progression compared to either treatment alone (Figures 7B–7D), suggesting a synergistic effect between the two agents.

To elucidate the underlying mechanisms, we processed tumor tissues for ELISA analysis, which revealed that UNC0379 treatment increased intratumoral levels of IFN-β (Figure 7E). Flow cytometry analysis provided additional insights, demonstrating that the combination of UNC0379 and anti-PD-1 led to a marked increase in the infiltration of both CD8^+^ T cells and NK cells into the tumor microenvironment (Figures 7F–7H). These immune cells play crucial roles in mediating tumor cell destruction, and their increased abundance suggests that UNC0379 enhances the immunomodulatory effects of anti-PD-1 therapy.

Taken together, these findings highlight the potential of KMT5A inhibition as a therapeutic strategy for CRC. Although UNC0379 monotherapy exhibited limited efficacy, its use as an adjuvant to anti-PD-1 therapy significantly improved antitumor outcomes.

## Discussion

IRF3 is a pivotal transcription factor (TF) known for its role in regulating immune responses and promoting apoptosis, which together contribute to the inhibition of tumor cell survival. In response to cellular stress or infection, tumor cells can activate the cGAS/STING/TBK1/IRF3 signaling pathway. This activation leads to sustained production of IFN-I, crucial mediators of the immune response, ultimately causing cancer cell growth arrest and triggering apoptosis.^24^ Despite IRF3’s central role in antitumor immunity, its activation is often downregulated in tumor cells through various mechanisms, which allows the cancer cells to evade immune surveillance.^25^^,^^26^^,^^27^^,^^28^ Our research uncovered a new regulatory mechanism by which IRF3 function is controlled. Specifically, we found that the lysine methyltransferase KMT5A interacts with IRF3 in the cytoplasm and monomethylates it, leading to an inhibition of IRF3 phosphorylation and subsequent activation. Phosphorylation is a critical PTM that allows IRF3 to become fully activated and translocate to the nucleus to drive IFN-I gene expression. The methylation by KMT5A effectively blocks this activation, revealing a novel crosstalk between methylation and phosphorylation in the regulation of IRF3. The overexpression of KMT5A in tumor cells results in the lysine methylation of IRF3, thereby diminishing its role in activating antitumor immunity and promoting immune evasion.

Although extensive studies have highlighted the importance of IRF3 phosphorylation and dephosphorylation in modulating its function,^5^^,^^29^^,^^30^^,^^31^^,^^32^^,^^33^ the role of other PTMs, such as methylation, in regulating IRF3 activity is less understood. Previous research has shown that the enzyme NSD3 can catalyze the mono-methylation of IRF3 at lysine 366 (K366), playing a critical role in antiviral innate immunity.^12^ However, the significance of IRF3 methylation in cancer has remained unclear. Our findings indicate that IRF3 is methylated at lysine 193 (K193) through its interaction with KMT5A, which suppresses the production of IFN-β, a key cytokine in antitumor immunity. This methylation at K193 is essential for tumor survival, illustrating the diverse biological effects that different PTMs can impart on the same protein. Notably, the K193 site of IRF3 is conserved across species, such as mice, pigs, cows, and monkeys. However, whether KMT5A can methylate IRF3 at this site in species other than humans and mice remains to be further explored. Additionally, other lysine residues on IRF3 may also be subject to methylation by KMT5A, necessitating more comprehensive studies on the full spectrum of KMT5A-mediated IRF3 regulation.

Protein lysine methylation is catalyzed by a family of enzymes known as protein lysine methyltransferases (PKMTs), and dysregulation of these enzymes has been linked to various malignancies.^34^ KMT5A is a well-characterized lysine methyltransferase known to methylate substrates, such as H4K20, SNIP1 K30, and CD147 K234.^18^^,^^19^^,^^35^^,^^36^ Studies have shown that KMT5A plays significant roles in tumor metastasis, DNA damage repair, and inflammatory responses.^18^^,^^37^^,^^38^ It is well established that KMT5A regulates gene expression by modifying histone H4K20me^39^ and can also function as a cofactor in antiviral immunity through activation of IRF7.^40^ In cancer progression, KMT5A often acts as an oncogene.^41^^,^^42^^,^^43^ Our findings suggest that KMT5A regulates IRF3 not at the transcriptional or translational level, but post-translationally, by inhibiting its phosphorylation while maintaining IRF3 protein levels. This is consistent with studies showing that KMT5A overexpression in CRC correlates with poor patient prognosis, as observed in other cancers.^44^ Therefore, KMT5A represents a promising therapeutic target in CRC.

The dynamic interplay between methylation and phosphorylation is a recurring theme in PTM regulation. TFs are frequently modified by multiple types of PTMs, which fine-tune their activity. Our research demonstrates that KMT5A overexpression promotes mono-methylation of IRF3 at K193, thereby inhibiting its phosphorylation and subsequent activation. Notably, there is an inverse correlation between IRF3 K193 methylation and its phosphorylation status. By methylating IRF3, KMT5A reduces the production of IFN-I, which diminishes the infiltration of immune cells such as CD8^+^ T cells into the tumor microenvironment. In our study, we observed that UNC0379 can also enhance NK cell infiltration, although further research is required to validate and elucidate this finding. This reduced immune cell infiltration promotes an immune-privileged state that facilitates tumor progression.

In conclusion, we have identified a novel regulatory mechanism of IRF3 involving methylation as a PTM, and we demonstrated that IRF3 is a direct substrate of KMT5A. KMT5A-mediated mono-methylation of IRF3 at K193 plays a critical role in CRC immune evasion by suppressing IRF3 phosphorylation and inhibiting the production of IFN-I. To further validate these findings, we employed UNC0379, a selective competitive inhibitor of KMT5A, which disrupted the interaction between KMT5A and IRF3. In both *in vitro* and *in vivo* experiments, UNC0379 was shown to promote IFN-β production and suppress tumor cell growth. Although UNC0379 alone exhibited limited therapeutic efficacy, its combination with anti-PD1 therapy significantly enhanced the immune response, suggesting that KMT5A inhibition could improve the outcomes of CRC immunotherapy. These findings propose UNC0379 as a potential adjuvant therapeutic strategy to boost the efficacy of immune checkpoint inhibitors in treating CRC, providing new insights into the complex regulatory network governing innate immunity and its potential implications in cancer biology.

### Limitations of the study

This study uncovers a novel molecular function of the KMT5A-IRF3 axis in mediating tumor immune evasion. Nevertheless, several limitations exist in this work. On one hand, direct evidence supporting the detailed molecular mechanism is inadequate. On the other hand, systematic profiling of the tumor immune microenvironment is insufficient.

We have confirmed that the methylation level of IRF3 at lysine 193 is negatively correlated with its phosphorylation level, and KMT5A inhibits IRF3 activation by catalyzing methylation at this residue. However, the precise molecular mechanism by which this methylation modification blocks IRF3 phosphorylation remains unclear. Apart from IRF3, KMT5A can modulate immune responses through other substrates such as histone H4K20. Although the K193R mutant was applied to verify the specificity of this modification site, the presence of other parallel regulatory pathways cannot be completely excluded.

Furthermore, this research only centered on CD8^+^ T cells and NK cells, while the roles of other immune cells, including macrophages, regulatory T cells, and neutrophils in the KMT5A-IRF3 pathway have not been investigated. In addition, although male and female subjects were randomly selected throughout all experiments, we did not further dissect the potential impact of sex on experimental outcomes. Further in-depth and comprehensive research is required to resolve all the above unresolved issues.

## Resource availability

### Lead contact

Further information and requests for resources and reagents should be directed to and will be fulfilled by the lead contact, Li Sun (litchisun@163.com).

### Materials availability

This study did not generate new unique reagents and all materials in this study are commercially available.

### Data and code availability

• Data: the data reported in this paper are available in deposited data in the key resources table. RNA-seq data analysis were available at GEO database (accession: GSE312287) and NCBI SRA database (accession: NCBI SRA:PRJNA1372776). Mass spectrometry data are available at ProteomeXchange (PXD: PXD073621).
• Code: no original code was generated in this study. All software and algorithms used are described in the STAR Methods and the key resources table.
• Any additional information required to reanalyze the data reported in this paper is available from the lead contact.

## Acknowledgments

This work was supported by 10.13039/501100012166National Key Research and Development Program of China (2023YFC3402102, 2022YFA1105303), The 10.13039/501100001809National Natural Science Foundation of China (82425041, 82330084, 82273254, 82403349, 82403387, 82504215, and 82503378), 10.13039/501100002858China Postdoctoral Science Foundation (2023M741273 and 2024T170308), Chutian Talent Program of Hubei Province (SCZ202409), 10.13039/501100003819Natural Science Foundation of Hubei Province (2021CFA006, 2024AFB048), and Basic Research Support Program of Huazhong University of Science and Technology (2023BR036).

## Author contributions

L.S. conceived and supervised the entire project and acquired funding; P.L., J.H., and G.W. contributed to the experimental design; P.L. performed the majority of the experiments, including animal studies, western blot, ELISA, and led the RNA-seq data analysis with assistance from Q.W., X.S., M.L., and Z.R.; mass spectrometry analysis was conducted collaboratively by P.L. and C.H.; C.Y. and R.X. were responsible for the CD8^+^ T cell dependence tests; clinical samples were provided by A.L. and L.L.; the manuscript was primarily drafted by P.L., C.Y., and L.S. with critical input from all authors; J.H. provided additional proofreading; all authors have read and agreed to the published version of the manuscript.

## Declaration of interests

The authors declare no competing interests.

## STAR★Methods

### Key resources table

**Table:** 

REAGENT or RESOURCE	SOURCE	IDENTIFIER
**Antibodies**		

Phospho-IRF3-S386 Rabbit mAb	ABclonal	CAT#AP0623; RRID: AB_2771210
IRF3 Rabbit pAb	ABclonal	CAT#A2172SP; RRID: N/A
IRF3 Monoclonal antibody	Proteintech	CAT#66670-1-Ig; RRID: AB_2882024
KMT5A Rabbit mAb	ABclonal	CAT#A4136; RRID: AB_2863193
MDA5 Rabbit mAb	ABclonal	CAT#A2419; RRID: AB_3674625
Rig-I/DDX58 Rabbit mAb	ABclonal	CAT#A22478; RRID: N/A
DDDDK-Tag Rabbit mAb	ABclonal	CAT#AE092; RRID: AB_2940847
Rabbit anti HA-Tag pAb	ABclonal	CAT#AE036; RRID: AB_2771924
HA-Tag (C29F4) Rabbit mAb	Cell Signaling Technology	CAT#3724; RRID: AB_1549585
Mouse anti His-Tag mAb	ABclonal	CAT#AE003; RRID: AB_2728734
Pan Mono-Methyl lysine Rabbit pAb	ABclonal	CAT#A18293; RRID: AB_2862066
Di-Methyl Lysine Motif MultiMab® Rabbit mAb mix	Cell Signaling Technology	CAT#14117; RRID: AB_2798396
Tri-Methyl Lysine Motif Rabbit mAb	Cell Signaling Technology	CAT#14680; RRID: AB_2798568
GAPDH Rabbit pAb	ABclonal	CAT#AC027; RRID: AB_2769572
Vinculin Polyclonal antibody	Proteintech	CAT#26520-1-AP; RRID: AB_2868558
PE anti-mouse CD45 Antibody	BioLegend	CAT#147711; RRID: AB_2563597
APC anti-mouse CD3 Antibody	BioLegend	CAT#100236; RRID: AB_2561456
PerCP/Cyanine5.5 anti-mouse CD45 Antibody	BioLegend	CAT#147706; RRID: AB_2563538
BD Pharmingen™ PE-Cy™7 Rat Anti-Mouse CD8a	BD Pharmingen	CAT#552877; RRID: N/A
PE-conjugated anti-mouse CD45 Antibody	BioLegend	CAT#147712; RRID: AB_2563598
PE/Cyanine7 anti-mouse NK-1.1 Antibody	BioLegend	CAT#156513; RRID: AB_2888852

**Bacterial and virus strains**		

pLVX-U6-KMT5A(human)-shRNA1-PGK-EGFP-Puro	This paper	NA
pLVX-U6-KMT5A(human)-shRNA2-PGK-EGFP-Puro	This paper	NA
pLVX-U6-KMT5A(mouse)-shRNA1-PGK-EGFP-Puro	This paper	NA
pLVX-U6-KMT5A(mouse)-shRNA2-PGK-EGFP-Puro	This paper	NA

**Biological samples**		

human colorectal cancer tissue samples and paired adjacent non-cancerous tissues	Tongji hospital	NA

**Chemicals, peptides, and recombinant proteins**		

Poly (I:C):Kanamycin (1:1)	MedChemExpress	CAT#HY-107202A
UNC0379	MedChemExpress	CAT#HY-12335
Anti-HA Magnetic Beads	MedChemExpress	CAT#HY-K0201
Anti-Flag magnetic beads	MedChemExpress	CAT#HY-K0207
Anti-His magnetic beads	MedChemExpress	CAT#HY-K0209
Protein A/G magnetic beads	MedChemExpress	CAT#HY-K0202
Anti-mouse PD-1(CD279)-In Vivo	Selleck	CAT#A2122

**Critical commercial assays**		

Mouse IFN-beta ELISA kit	ABclonal	RK00420
Human Interferon Beta ELISA Kit	ABclonal	RK01630
Renilla-Firefly Luciferase Dual Assay Kit	MedChemExpress	HY-K1013
Subcellular Structure Nuclear and Cytoplasmic Protein Extraction Kit	BOSTER	AR0106

**Deposited data**		

Raw and analyzed data	This paper	the TCGA datasets (https://xenabrowser.net/datapages/)
RNAseq data	This paper	Sbmitted to GEO GEO:GSE312287 NCBI SRA:PRJNA1372776
Mass spectrometry data	This paper	Sbmitted to ProteomeXchange PXD: PXD073621

**Experimental models: Cell lines**		

Human: HEK-293T	ATCC	CBP60439
Human: RKO	ATCC	CBP60006
Human: HCT116	ATCC	CBP60028
Mouse:MC38	ATCC	CBP60825

**Experimental models: Organisms/strains**		

C57BL/6 mice	Vital River Laboratory Animal Technologies	NA

**Oligonucleotides**		

shKMT5A#1(human)	MIAOLING PLASMID	5 ′-GAGGGCCTATTTCCCATGA-3′
shKMT5A#2(human)	MIAOLING PLASMID	5′GAGGGCCTATTTCCCATGA-3′
shKMT5A#1(mouse)	MIAOLING PLASMID	5′-GTGGCTGCTACATGTACTATTT-3′
shKMT5A#2(mouse)	MIAOLING PLASMID	5′-GAGGAACACCGGGAACGTTATA-3′
Human IRF3 sgRNA#1	tsingke	5′- AGCTCGAATTGCGTCAACCC-3′
Human IRF3 sgRNA#2	tsingke	5′- AGCGTGTTGACCTGTGTGGC -3′
Primers for IFN-beta1b(forward)	tsingke	5′-ATGACCAACAAGTGTCTCCTCC-3′
Primers for IFN-beta1b(reverse)	tsingke	5′-GGAATCCAAGCAAGTTGTAGCTC-3′
Primers for KMT5A(forward)	tsingke	5′-ACCGACGGGGAGAACGTATT-3′
Primers for KMT5A(reverse)	tsingke	5′-GCATTCCAGAGCATTTGTTCG-3′

**Recombinant DNA**		

pLV2-CMV-IRF3(human)-FLAG-Puro	Miaoling plasmid	P70281
pCMV-3×HA-KMT5A(human)-SV40-Neo	Miaoling plasmid	P36496
IFN-Beta_pGL3	Miaoling plasmid	p5314
pRL-TK	Miaoling plasmid	p0372

**Software and algorithms**		

Prism	GraphPad Software	https://www.graphpad.com/features
ImageJ	NIH	https://github.com/imagej/ImageJ
PyMOL	schrodinger.	https://www.schrodinger.com/platform/products/pymol/
FlowJo	BD Biosciences	https://flowjo.com
Xcalibur 2.2	ThermoFisher Scientific	https://so.sengfeng.cn/s/xcalibur-lc-ms
GSEA v4.3.2	N/A	https://www.gsea-msigdb.org/gsea/index.jsp

### Experimental model and study Participant Details

#### Cell line and cell culture

The human embryonic kidney cell line HEK293T, human CRC cell lines (RKO and HCT116), and mouse colon cancer cell line MC38 were obtained from the American Type Culture Collection (ATCC). HEK293T, RKO, and MC38 cells were cultured in DMEM basal medium. HCT116 cells were maintained in RPMI-1640 medium. 10% fetal bovine serum, 1% penicillin, and 1% streptomycin were added to DMEM and RPMI-1640 medium. Cells were cultured in a humidified incubator maintained at 37°C with 5% CO_2_. All Cell lines STR (Short Tandem Repeat) identification has been conducted. All cell lines were free of mycoplasma contamination.

#### Animals

All animal experiments in this study received approval from the Animal Care and Use Committee of Tongji Hospital (Ethical Approval No.: TJH-201904007). The sample sizes were justified by statistical considerations and statistical power analyses. Mice were randomly divided into groups. The assignment was blinding during the experiment and outcome assessment.The 6-8-week-C57BL/6 mice used in the experiments were obtained from Vital River Laboratory Animal Technologies (Beijing, China). Male and female mice were randomly selected in all experiments to ensure no statistically significant difference in sex ratio. All mice were housed and managed under standard specific pathogen-free (SPF) conditions in the animal facility.

#### Human samples

All clinical specimens included in this study were collected from Wuhan Tongji Hospital. The study protocol was approved by the Ethics Committee of Huazhong University of Science and Technology and the Institutional Ethics Committee of Wuhan Tongji Hospital. All experimental procedures were conducted in strict accordance with relevant national regulations and standard guidelines. A total of 12 patients diagnosed with colorectal cancer were enrolled, consisting of six male and six female subjects. All patients were fully informed of the study content and provided written informed consent prior to sample collection.

### Method details

#### Plasmid construction

The Flag-IRF3, HA-KMT5A, IFN-Beta-Pgl3 and pRL-TK were purchased from MiaoLing Biology (Wuhan, China). Subsequently, the IRF3 mutants (K193R, K366R) were generated from the Flag-IRF3 plasmid by a site-directed mutagenesis kit (C214-01; Vazyme). The lentiviral plasmids pLVX-IRF3-puro and pLVX-KMT5A-neo were constructed by cloning IRF3 and KMT5A genes into the pLVX-puro/*neo* vector. The lentiviral plasmids pLV2-U6-KMT5A-puro were constructed by inserting the KMT5A shRNA into the pLV2-U6-puro vector. The shRNA sequences are mentioned in the key resources table above.

#### Stable knockdown and overexpressing cell lines

Stable knockdown cell lines were generated through lentiviral transduction. The lentiviruses were produced by co-transfecting HEK293T cells with plasmids (pLV2-U6-IRF3-puro or pLV2-U6-KMT5A-puro, PMD2.G, and psPAX2). In this way, lentiviruses for the overexpression IRF3 and KMT5A were generated. Following infection, cells were treated with 2.0 μg/mL puromycin for 48 h to select for transduced cells. Successful cell line construction was confirmed by immunoblot assays.

#### Establishment of knockout cell line using CRISPR/Cas9

IRF3 knockout cells were generated using the CRISPR/Cas9 system. Single-guide RNAs were designed using the public E-CRISP network (http://www.e-crisp.org/E-CRISP/) and cloned into either the PX462 (Addgene #62987) or PX459 (Addgene #62988) plasmid. After transfection of the plasmids into cells for 24 h, cells were selected with puromycin for 48 h. Single cells were then cultured in 96-well plates for 2 weeks before being transferred to 12-well plates. KO cells were validated by immunoblotting. The sgRNA sequences are mentioned in the key resources table above.

#### Mass spectrometry analyses

HEK293T cells expressing either Flag-IRF3 or a control vector were lysed using NP-40 buffer, followed by immunoprecipitation (IP) with anti-Flag magnetic beads. The Flag-IRF3 and associated proteins were eluted using Flag peptides (100 μg/mL) and subsequently resolved via sodium dodecyl sulfate-polyacrylamide gel electrophoresis (SDS-PAGE), followed by Coomassie brilliant blue staining.

For mass spectrometry analysis, the stained gels were sent to the Protein Chemistry and Proteomics Facility at the Technology Center of Harbin Institute of Technology for Protein Research. Eluted proteins were precipitated using 0.1 M ammonium acetate in 100% methanol. Following reduction and alkylation (5 mM dithiothreitol, 10 mM iodoacetamide), the samples underwent overnight digestion with sequencing-grade trypsin. Peptide mixtures were vacuum-dried using a SpeedVac concentrator and re-suspended in solvent A (water with 0.1% formic acid) prior to liquid chromatography-tandem mass spectrometry (LC-MS/MS) analysis.

The peptide mixtures were then separated using a Thermo Dionex Ultimate 3000 HPLC system coupled directly to an Orbitrap Q Exactive mass spectrometer (Thermo Fisher Scientific, Bremen, Germany), operating in data-dependent acquisition mode, and the data were acquired using Xcalibur 2.2 software.

#### Western blot and immunoprecipitation

The indicated cells were lysed using NP-40 lysis buffer for 30 min at 4°C, followed by protein concentration measurement using a bicinchoninic acid (BCA) assay kit (Thermo Fisher Scientific). Proteins were mixed with 5× protein sample buffer [250 mM Tris-HCl (pH 6.8), 10% SDS, 30% glycerol, 5% β-mercaptoethanol, and bromophenol blue], and samples were boiled at 100°C for 5 to 10 min. Proteins were then separated by electrophoresis on an 8 to 12% pre-cast SDS-polyacrylamide mini-gel and transferred to a polyvinylidene difluoride (PVDF) membrane. Membranes were incubated with the indicated primary antibodies overnight at 4°C, followed by incubation with HRP-conjugated secondary antibodies for 2 h at room temperature. Chemiluminescence detection was used to analyze the membranes. For immunoprecipitation (IP), magnetic beads were washed three times with NP-40 lysis buffer, then incubated overnight at 4°C with primary antibodies (HY-K0205,MCE). The beads were subsequently rotated with the cell lysate, and immunoblot assays were performed.

#### Cytoplasmic and nuclear protein extraction

Briefly, cells in the culture were collected using a cell scraper and washed twice with ice-cold PBS. Cytoplasmic and nuclear protein extracts were then prepared from the cells using a Nuclear and Cytoplasmic Protein Extraction Kit (Boster, China) according to the manufacturer’s instructions. Protein concentrations were determined using a BCA Protein Assay Kit (Boster, China), and the samples were stored at −80 °C until use.

#### Clinical samples preparation

Clinical samples and patients’ information were obtained from Wuhan Tongji Hospital. This study was approved by the Ethics Committee of the Huazhong University of Science and Technology and the Institutional Ethics Committeeof Tongji Hospital. All patients were fully informed of the study content and provided written informed consent prior to sample collection. All experimental procedures were conducted in strict accordance with relevant national regulations and standard guidelines. A total of 12 patients diagnosed with colorectal cancer were enrolled, consisting of six male and six female subjects.Frozen tissue specimens were cryogenically pulverized in liquid nitrogen using pre-chilled mortars to achieve a fine powder consistency. The homogenized tissue was then lysed in NP-40 buffer (1% NP-40, 150 mM NaCl, 50 mM Tris-HCl pH 7.4, supplemented with protease/phosphatase inhibitors) at a 1:10 weight/volume ratio. Ultrasonic disruption was performed with a 20 kHz probe under strict temperature control (4°C ice-water bath), employing three intermittent 10-s pulses (30-s cooling intervals between pulses) at 60% amplitude to prevent protein denaturation. Following centrifugation at 12,000×g for 15 min at 4°C, the supernatant was collected for protein quantification using BCA assay, with homogenization efficiency verified microscopically to ensure complete tissue disaggregation prior to downstream analyses.

#### *In vitro* methylation assays

Recombinant proteins for KMT5A (HY-P74613, MedChemExpress) and His-tagged IRF3 (HY-P702766, MedChemExpress) were obtained from the indicated suppliers. For *in vitro* methylation assays, 1 μg of KMT5A was combined with 5 μL of 5× PKMT buffer [10 mM Tris-HCl (pH 8), 2% glycerol, 0.8 mM KCl, and 1 mM MgCl2], 13 μM S-adenosyl-L-methionine, His-IRF3 purified protein or biotin-tagged PD-L1 peptides as substrates, and H2O, to a final volume of 25 μL. The reaction mixture was incubated at 37°C for 10 h. The reaction was terminated by adding 5× protein sample buffer [250 mM Tris-HCl (pH 6.8), 10% SDS, 30% glycerol, 5% β-mercaptoethanol, and bromophenol blue], followed by boiling the samples at 95°C for 10 min. His-tag magnetic beads were washed three times with NP-40 buffer and then incubated with a mixture of His-tagged IRF3 protein and purified KMT5A protein overnight at 4°C with constant rotation. The magnetic beads were then washed five times with wash buffer and subjected to immunoblot assays.

#### Quantitative Real-Time polymerase chain reaction (qRT-PCR)

Total RNA was extracted from the indicated cells using TRIzol reagent (Invitrogen, Carlsbad, CA, USA) following the manufacturer’s protocol. Complementary DNA (cDNA) was synthesized using the ABScript III RT Master Mix for quantitative polymerase chain reaction (qPCR) with a gDNA Remover kit (RK20429, ABclonal). Quantitative real-time PCR was then performed using the ABScript III RT Master Mix for qPCR (RK20429, ABclonal) on a QuantStudio 3 Real-Time PCR System (Thermo Fisher Scientific). The primers used were mentioned in the key resources table above.

#### T cell–mediated tumor cell killing assay

Dendritic cells (DCs) were isolated from peripheral blood mononuclear cells (PBMCs) and co-cultured with the indicated cancer cell lines. Pretreated DCs were then used to activate PBMCs. To analyze T cell-mediated tumor cell killing, the pretreated PBMCs were re-stimulated with IL-2 (100 IU/mL), anti-CD3 antibody (0.1 μg/mL), and anti-CD28 antibody (0.1 μg/mL). The indicated target cells were co-cultured with activated primary human T cells at a 10:1 ratio (effector:target). After 2 days of co-culture, T cells and cellular debris were removed by washing three times with phosphate-buffered saline (PBS). The remaining cells were fixed with 4% paraformaldehyde at room temperature for 15 min and subsequently stained with crystal violet. The optical density at 570 nm was quantified using a spectrophotometer to assess the extent of tumor cell killing.

#### Reporter gene assays

Cells were cultured in 24-well plates and transfected with the specified plasmids. To normalize transfection efficiency, the pRL-TK (Renilla luciferase) reporter plasmid was co-transfected into each well, and an empty vector plasmid was used to ensure consistent total DNA amounts across all wells. After 24 h, in some experiments, cells were either stimulated with poly(I:C) or left unstimulated for 12 h. Following stimulation, cells were harvested and analyzed using a dual-luciferase assay kit. Firefly luciferase activity was normalized to Renilla luciferase activity. All reporter gene assays were conducted in triplicate.

#### Murine tumor models and treatments

All animal experiments in this study received approval from the Animal Care and Use Committee of Tongji Hospital, The indicated MC38 cells (2 × 10^5^) were subcutaneously injected into 6-week-old mice. Mice were randomized prior to the initiation of treatment, and outcome analyses were performed in a blinded manner. In all experiments, when the average tumor volume reached approximately 50 mm^3^, treatment was initiated with either anti-mouse PD-1 antibody (100 μg/mouse) or UNC0379 (3 mg/kg), administered once every three days. Tumor volumes were measured at specified time points using a vernier caliper and calculated according to the formula: 0.5 × L × D^2^ (L: longest diameter; D: shortest diameter). After euthanasia, subcutaneous tumors were excised and weighed, and the tissues were subjected to further analysis.

#### Preparation of single-cell suspension

Subcutaneous tumors from the mice were cut into small pieces and incubated in 3 mL of serum-free RPMI-1640 medium containing collagenase IV (50 μL of 25 mg/mL;, V900893, Sigma-Aldrich), hyaluronidase (50 μL of 32 mg/mL, H3506, Sigma-Aldrich), and DNase I (25 μL of 10 mg/mL, 10104159001, Roche) at 37°C in a shaking incubator (150 rpm) for 1 h. After complete enzymatic dissociation, 7 mL of serum-free RPMI-1640 medium was added to the tube to dilute the enzyme concentration. Following filtration and centrifugation, 1 mL of ACK lysis buffer was added to the tube for 1 min to lyse red blood cells, followed by neutralization. For subsequent staining experiments, the samples were resuspended and kept on ice.

#### Flow cytometry analysis

Single-cell suspensions from subcutaneous tumors were blocked with anti-mouse CD16/32 antibody (101319, BioLegend) diluted 1:200 for 30 min. After washing and centrifugation, samples were stained with the Zombie UV Fixable Viability Kit (423106, BioLegend) to exclude dead cells. The following antibodies were then used for staining at 25°C in the dark for 30 min: PE-conjugated anti-mouse CD45 antibody (147711, BioLegend), APC-conjugated anti-mouse CD3 antibody (100236, BioLegend), PerCP/Cyanine5.5-conjugated anti-mouse CD45 antibody (147706, BioLegend), PE/Cy7-conjugated anti-mouse CD8 antibody (552877, BD Pharmingen), PE-conjugated anti-mouse CD45 antibody (147712, BioLegend), and PE/Cyanine7 anti-mouse NK-1.1 antibody (156513, BioLegend). Following staining, cells were resuspended in 500 μL phosphate-buffered saline (PBS) and filtered through a 35-μm mesh to remove aggregates prior to acquisition. Instrument calibration was performed using standardized calibration beads to ensure signal stability. Key instrument parameters were optimized as follows: forward scatter (FSC) voltage was adjusted to position the primary cell population within the 10^3^–10^4^ range, while side scatter (SSC) voltage was tuned to resolve granularity signals and discriminate cellular debris. Spectral overlap compensation was applied using single-stained controls to correct for fluorescence spillover between PE-Cy7 and APC channels. Data acquisition was performed at a flow rate ≤1,000 events per second to minimize swarm effects, with a minimum of 10^4^ events recorded per sample to ensure statistical robustness. All data were analyzed using FlowJo software (v10.8.1). The flow cytometry gating strategy for identifying CD8^+^ T cells and NK cells followed a sequential hierarchical approach. Initially, the lymphocyte population was gated based on forward scatter (FSC-A) and side scatter (SSC-A) properties. Subsequent pulse processing analysis of FSC-A versus FSC-H (or SSC-A versus SSC-H) was performed to exclude cell doublets, ensuring that only single, live cells were included in downstream analysis. Leukocytes were further confirmed and gated using CD45 staining.For CD8^+^ T cell identification, CD3^+^ T cells were first selected from the pre-gated single live leukocyte population. The CD8^+^ T cell subset was then identified as CD3^+^ CD8^+^ cells. For NK cell identification, CD3^+^ cells (primarily T cells) were excluded, and the remaining population was gated based on the expression of characteristic NK cell markers, NK1.1 (or CD49b) in mouse samples.

### Quantification and statistical analysis

Differences were analyzed using Student’s *t* test, and one-way analysis of variance (one-way ANOVA) was applied for comparisons across multiple groups. Gene Expression Omnibus (GEO) datasets were retrieved from the PubMed database hosted by the National Center for Biotechnology Information (NCBI; https://www.ncbi.nlm.nih.gov/). The online survival analysis platform Kaplan-Meier Plotter (http://kmplot.com/analysis/) was adopted for survival analyses. Gene set enrichment analysis was carried out with GSEA v4.3.2 software. All data processing and statistical analyses were performed using GraphPad Prism 9.0. All quantitative data were expressed as mean ± standard deviation (mean ± SD). Statistical significance was defined as *p* < 0.05. The significance markers were labeled as follows: ∗*p* < 0.05, ∗∗*p* < 0.01, ∗∗∗*p* < 0.001, ∗∗∗∗*p* < 0.0001.

## Supplemental information

Document S1. Figures S1–S7

Table S1. List of potential IRF3-binding proteins from mass spectrometry analysis

Table S2. List of differential mRNA from RNA-seq analysis of RKO with shKMT5A and shNC
