# Supplementary material for: KMT5A-mediated methylation of IRF3 promotes tumor progression through immune suppression
Source: iScience. 2026 Jul 21;29(8):116902. doi: 10.1016/j.isci.2026.116902 (PMC13392861; doi:10.1016/j.isci.2026.116902)
Supplement: Document S1. Figures S1–S7 [file mmc1.pdf]

**iScience, Volume *29***

## Supplemental information

# KMT5A-mediated methylation of IRF3 promotes tumor progression through immune suppression

**Pengcheng Li, Chengxin Yu, Runshi Xie, Changsheng Huang, Qi Wu, Anyi Liu, Xiaowei She, Mao Li, Zejun Rao, Lang Liu, Guihua Wang, Junbo Hu, and Li Sun**

# Figure S1

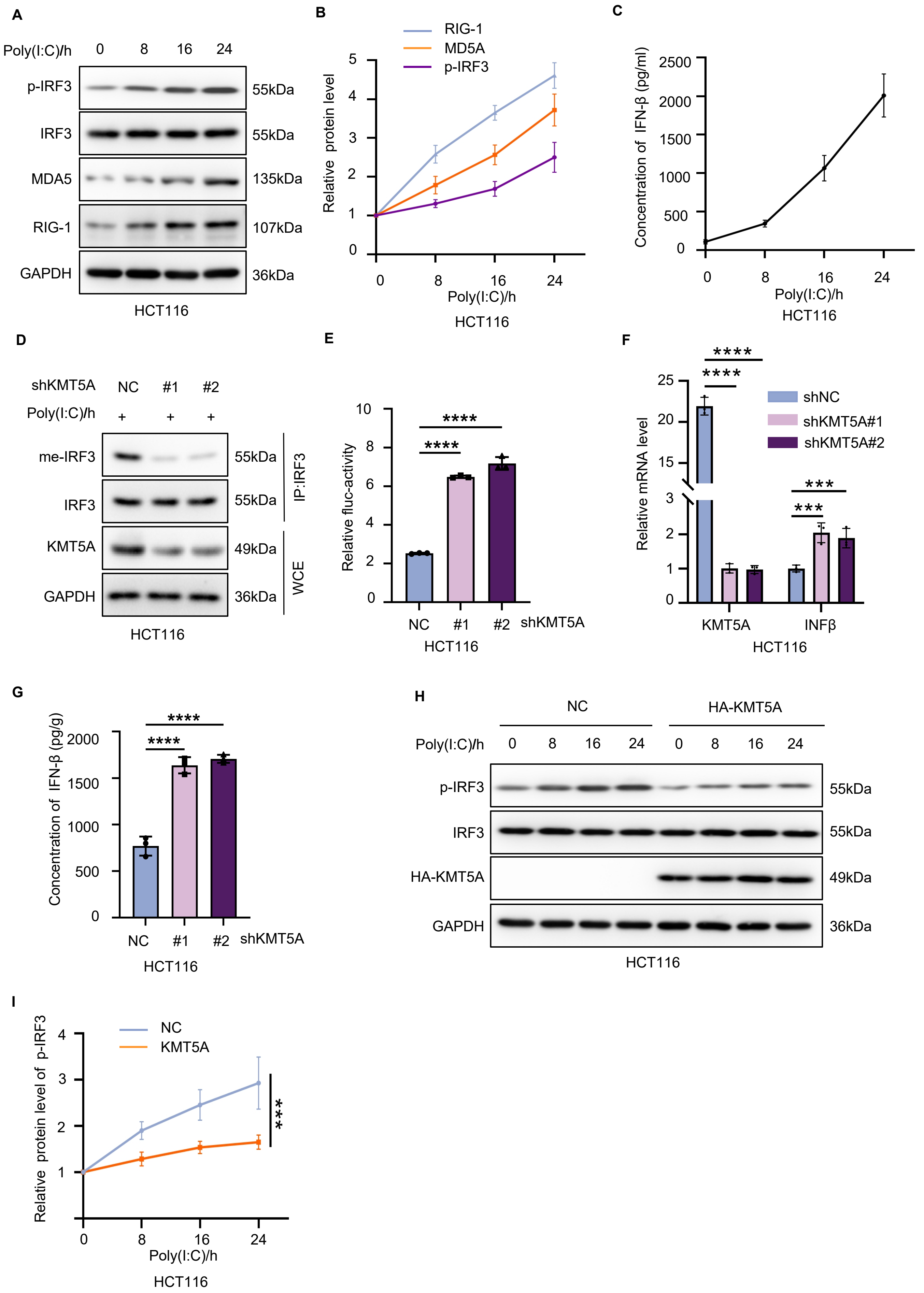
A
Poly(I:C)/h 0 8 16 24
p-IRF3 55kDa
IRF3 55kDa
MDA5 135kDa
RIG-1 107kDa
GAPDH 36kDa
HCT116
B
RIG-1
MD5A
p-IRF3
Relative protein level
Poly(I:C)/h
HCT116
C
Concentration of IFN-β (pg/ml)
Poly(I:C)/h
HCT116
D
shKMT5A NC #1 #2
Poly(I:C)/h + + +
me-IRF3 55kDa
IRF3 55kDa
KMT5A 49kDa
GAPDH 36kDa
IP:IRF3
WCE
HCT116
E
Relative fluc-activity
****
****
NC #1 #2 shKMT5A
HCT116
F
****
****
shNC
shKMT5A#1
shKMT5A#2
Relative mRNA level
***
***
KMT5A INFβ
HCT116
G
Concentration of IFN-β (pg/g)
****
****
NC #1 #2 shKMT5A
HCT116
H
NC HA-KMT5A
Poly(I:C)/h 0 8 16 24 0 8 16 24
p-IRF3 55kDa
IRF3 55kDa
HA-KMT5A 49kDa
GAPDH 36kDa
HCT116
I
NC
KMT5A
Relative protein level of p-IRF3
***
Poly(I:C)/h
HCT116

**Figure S1 Further validation in HCT116 cells of the inhibitory effect of KMT5A on IRF3 phosphorylation**

**A**: HCT116 cells were treated with Poly(I:C) and protein samples were collected at 0, 8, 16, and 24 hours for Western blot (WB) analysis. **B**: Quantification of p-IRF3, RIG-1, and MDA5 protein levels relative to control in the samples collected in A. **C**: IFN-β secretion levels at different time points from A were measured using ELISA. **D**: HCT116 cells transduced with control shRNA (shNC) or KMT5A-specific shRNAs (#1 and #2) were treated with Poly(I:C) and analyzed by WB using whole-cell lysates. **E**: HCT116 cells from D were co-transfected with the IFN-β-Luc reporter plasmid and pRL-TK plasmid, and after 24 hours, luciferase activity was measured using a dual-luciferase assay kit. **F**: The relative mRNA levels of KMT5A and INF-β in HCT116 cells from D were quantified using qPCR. **G**: IFN-β levels in the HCT116 cells from D were measured using ELISA. **H**: HCT116 cells, either wild-type or stably expressing HA-KMT5A, were treated with Poly(I:C) and whole-cell lysates were collected at 0, 8, 16, and 24 hours for WB analysis. **I**: Quantification of p-IRF3 protein levels relative to control in samples collected in **J**. For E, F and G statistical significance was determined using one-way ANOVA followed by Tukey's post-hoc test. For I, two-way ANOVA with Tukey's post-hoc test was used. Data are presented as mean $\pm$ s.d. Statistical significance is indicated as ***p < 0.001, ****p < 0.0001. All Western blot analyses were performed independently three times, yielding consistent results.

# figure S2

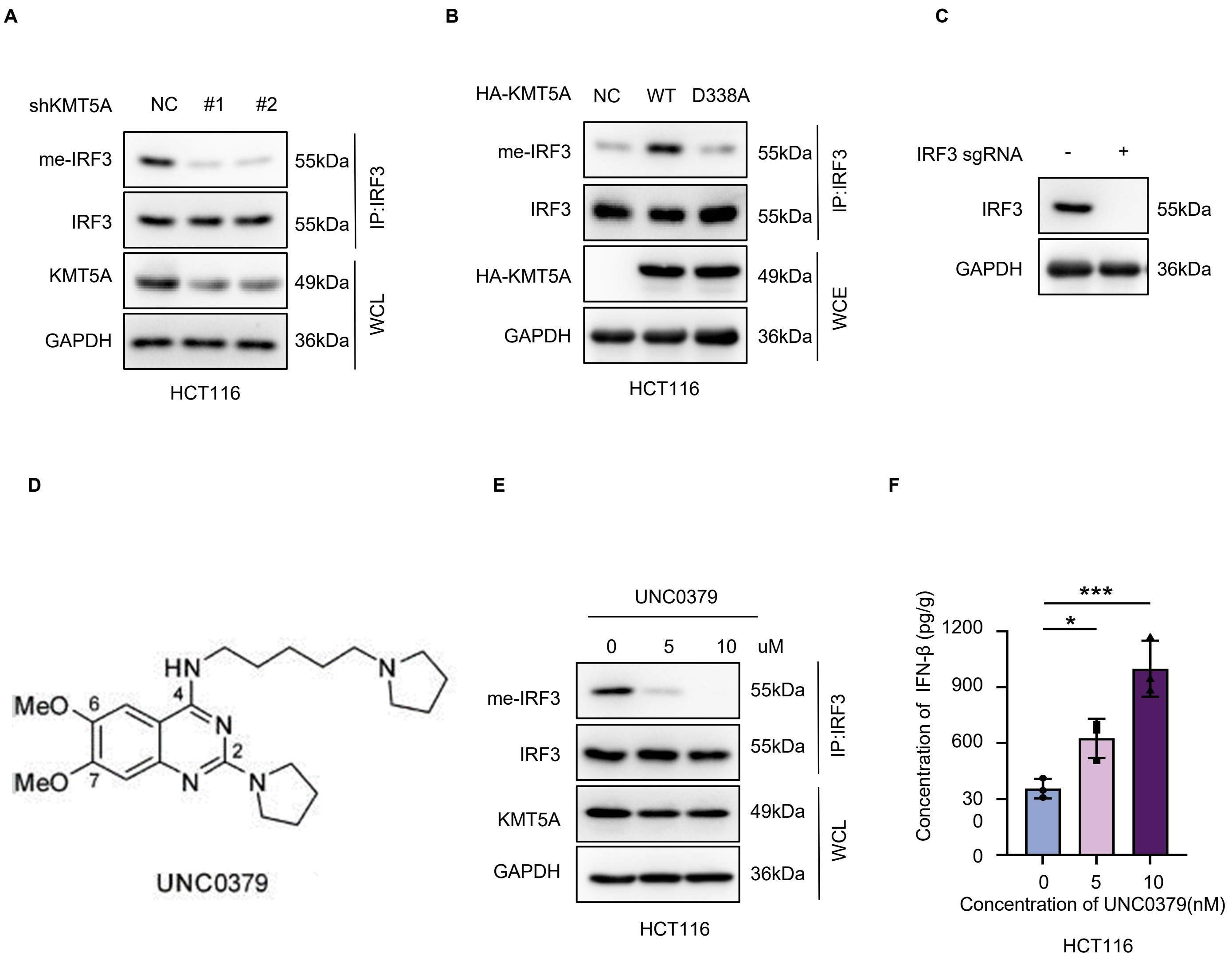


**Figure S2 Schematic chemical structure of UNC0379 and parallel validation assays in HCT116 cells**

A: Whole cell lysates were extracted from HCT116 cells with either control (shNC) or KMT5A shRNA (#1 and #2) silencing. IP was performed using anti-IRF3 antibodies, followed by WB analysis . B:HCT116 cells were transfected with HA-KMT5A WT or HA-KMT5A D338A, IP was performed using anti-IRF3 antibodies, followed by WB analysis. C:WCE from IRF3 WT and IRF3 KO RKO cells were collected for IB analysis. D:Chemical Structure of UNC0379. E: HCT116 cells were treated with either DMSO or varying concentrations of UNC0379. Immunoprecipitation (IP) was conducted using anti-IRF3 antibodies, followed by immunoblotting (IB) analysis. F: The levels of IFN-β in RKO cells from experiment E were quantified using ELISA. Data were analyzed using one-way ANOVA with Tukey‘s post-hoc test, presented as mean ± standard deviation (s.d.). Statistical significance was defined as *p < 0.05 and ***p < 0.001.

# figure S3

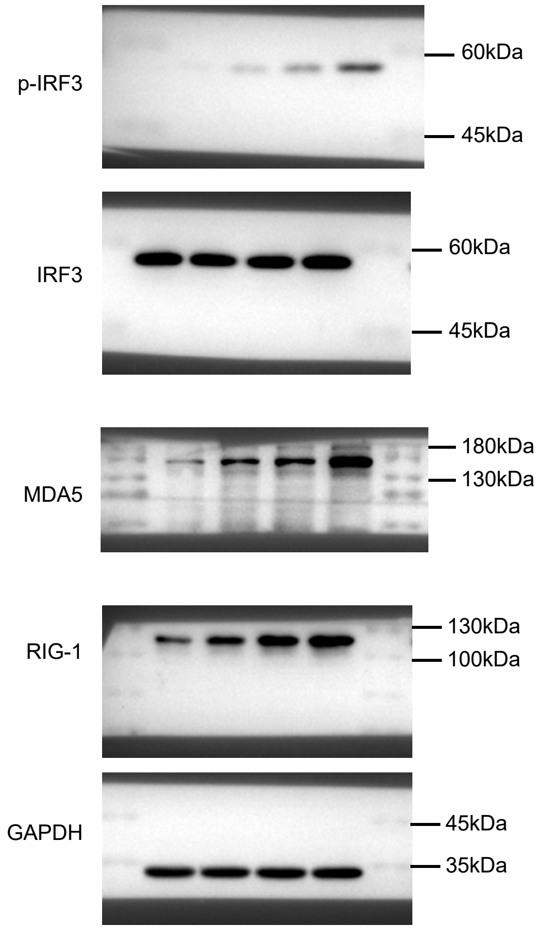


**Figure S3** Immunoblots corresponding to Figure 4A with molecular weight marker lanes.

## figure S4

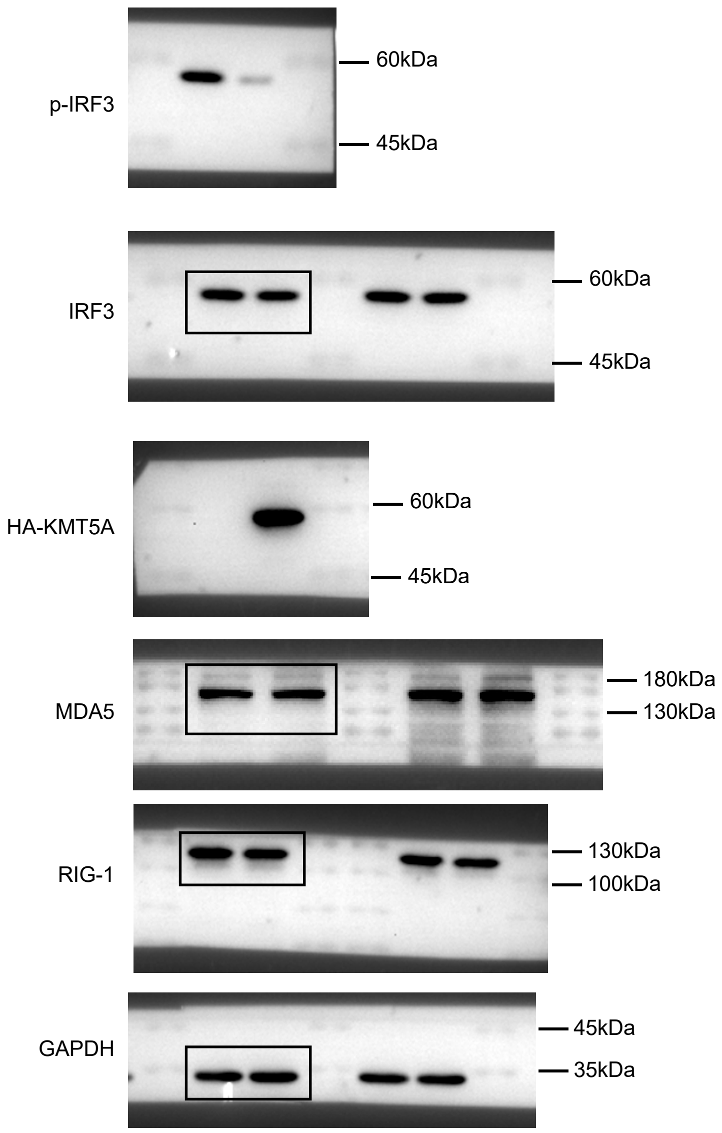


**Figure S4** Immunoblots corresponding to Figure 4D with molecular weight marker lanes.

## figure S5

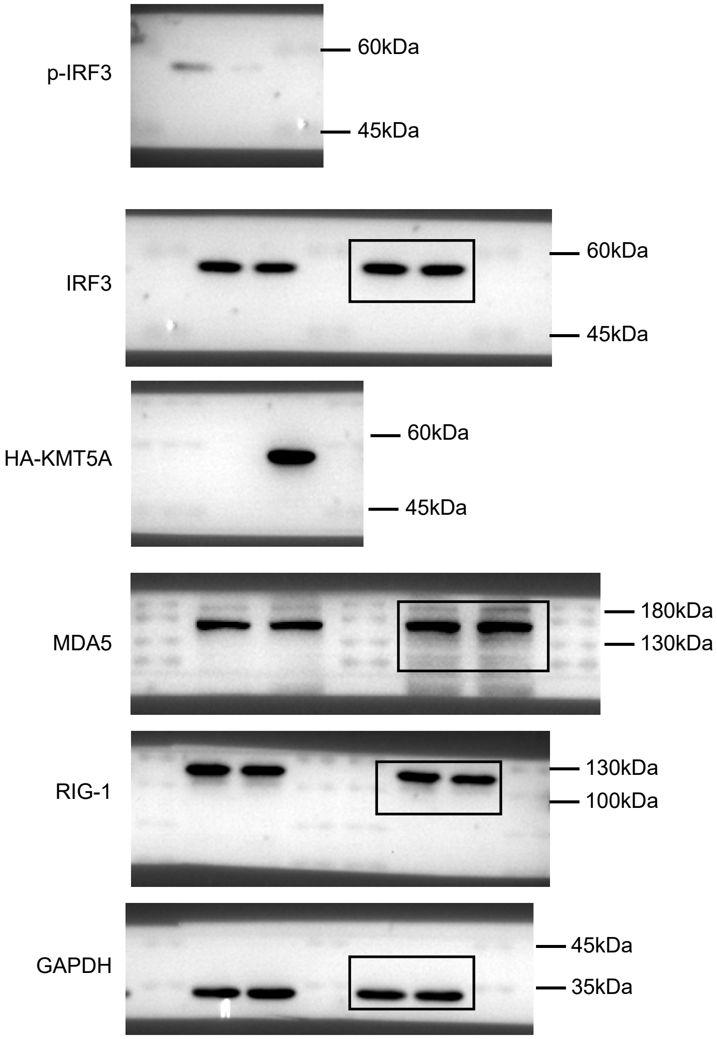


**Figure S5** Immunoblots corresponding to Figure 4E with molecular weight marker lanes.

**figure S6**

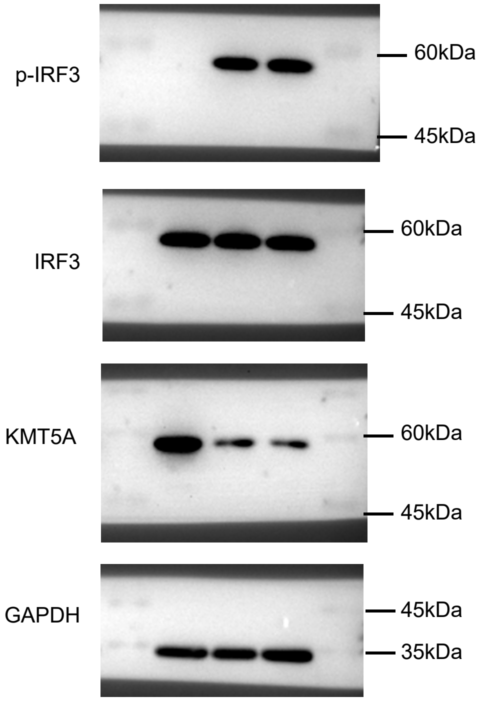


**Figure S6** Immunoblots corresponding to Figure 4F with molecular weight marker lanes.

figure S7

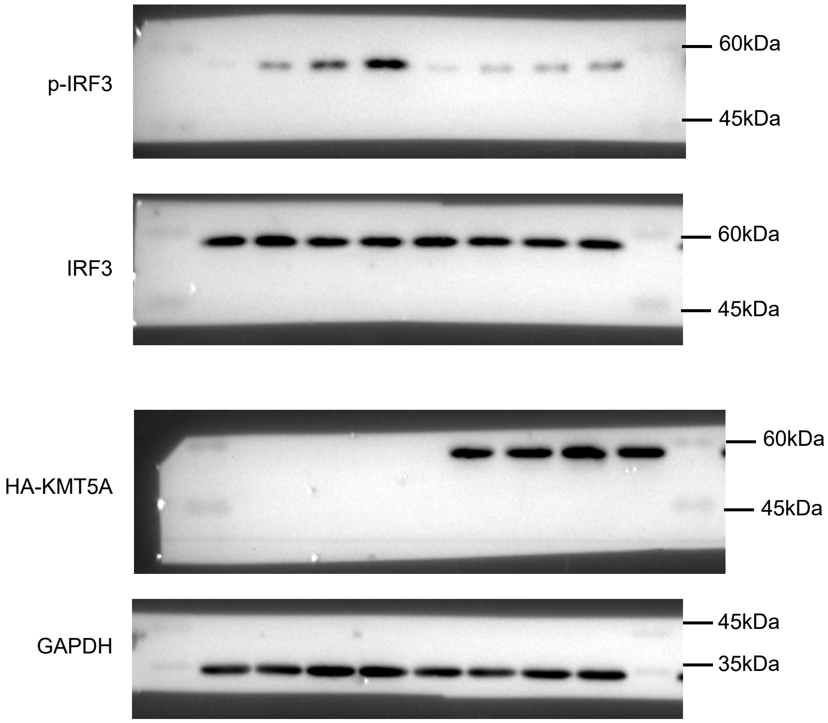


Figure S7 Immunoblots corresponding to Figure 4J with molecular weight marker lanes.
